# Performance of an Open-Source Large Language Model in Extracting Information from Free-Text Radiology Reports

**DOI:** 10.1148/ryai.230364

**Published:** 2024-05-08

**Authors:** Bastien Le Guellec, Alexandre Lefèvre, Charlotte Geay, Lucas Shorten, Cyril Bruge, Lotfi Hacein-Bey, Philippe Amouyel, Jean-Pierre Pruvo, Gregory Kuchcinski, Aghiles Hamroun

**Affiliations:** From the Department of Neuroradiology (B.L.G., A.L., C.B., J.P.P., G.K.), Department of Public Health (B.L.G., P.A., A.H.), and INclude Health Data Warehouse (C.G., L.S.), CHU Lille–Université Lille, Rue Emile Laine, 59000 Lille, France; Department of Radiology, UC Davis Health, Sacramento, Calif (L.H.B.); Université Lille, INSERM, CHU Lille, Institut Pasteur de Lille, U1167-RID-AGE - Facteurs de risque et déterminants moléculaires des maladies liées au vieillissement, Lille, France (P.A., A.H.); INSERM, U1172–LilNCog-Lille Neuroscience & Cognition, Université Lille, Lille, France (J.P.P., G.K.); and UAR 2014-US 41-PLBS–Plateformes Lilloises en Biologie & Santé, Université Lille, Lille, France (J.P.P., G.K.).

**Keywords:** Large Language Model (LLM), Generative Pretrained Transformers (GPT), Open Source, Information Extraction, Report, Brain, MRI

## Abstract

**Purpose:**

To assess the performance of a local open-source large language model (LLM) in various information extraction tasks from real-life emergency brain MRI reports.

**Materials and Methods:**

All consecutive emergency brain MRI reports written in 2022 from a French quaternary center were retrospectively reviewed. Two radiologists identified MRI scans that were performed in the emergency department for headaches. Four radiologists scored the reports’ conclusions as either normal or abnormal. Abnormalities were labeled as either headache-causing or incidental. Vicuna (LMSYS Org), an open-source LLM, performed the same tasks. Vicuna’s performance metrics were evaluated using the radiologists’ consensus as the reference standard.

**Results:**

Among the 2398 reports during the study period, radiologists identified 595 that included headaches in the indication (median age of patients, 35 years [IQR, 26–51 years]; 68% [403 of 595] women). A positive finding was reported in 227 of 595 (38%) cases, 136 of which could explain the headache. The LLM had a sensitivity of 98.0% (95% CI: 96.5, 99.0) and specificity of 99.3% (95% CI: 98.8, 99.7) for detecting the presence of headache in the clinical context, a sensitivity of 99.4% (95% CI: 98.3, 99.9) and specificity of 98.6% (95% CI: 92.2, 100.0) for the use of contrast medium injection, a sensitivity of 96.0% (95% CI: 92.5, 98.2) and specificity of 98.9% (95% CI: 97.2, 99.7) for study categorization as either normal or abnormal, and a sensitivity of 88.2% (95% CI: 81.6, 93.1) and specificity of 73% (95% CI: 62, 81) for causal inference between MRI findings and headache.

**Conclusion:**

An open-source LLM was able to extract information from free-text radiology reports with excellent accuracy without requiring further training.

**Keywords:** Large Language Model (LLM), Generative Pretrained Transformers (GPT), Open Source, Information Extraction, Report, Brain, MRI

*Supplemental material is available for this article.*

Published under a CC BY 4.0 license.

See also the commentary by Akinci D’Antonoli and Bluethgen in this issue.

## Graphical abstract

**Figure:**
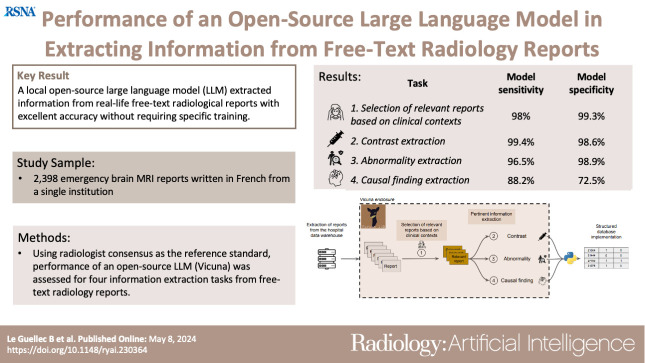

**Summary**

A local open-source large language model extracted information from real-life free-text radiology reports with excellent accuracy without requiring specific training.

**Key Points**

■ Vicuna (LMSYS Org), an on-premise and open-source large language model, reviewed 2398 emergency brain MRI free-text reports and achieved high performance metrics for detecting the presence of headache in the clinical context (F1 score: 98.0 [95% CI: 97.0, 98.7]), detecting the use of contrast medium injection in the protocol (F1 score: 99.6 [95% CI: 99.0, 99.9]), and study categorization as either normal or abnormal (F1 score: 97.3 [95% CI: 95.4, 98.6]).
■ Vicuna performed causal inference between a radiologic finding and a symptom with 82% accuracy, a task challenging even for radiologists (interrater κ < 0.80).

## Introduction

Scalable and flexible solutions to harness the potential of large unstructured radiology report databases are urgently needed (1). The expected “information revolution” (2) secondary to report structuring has yet to materialize, as large-scale retrospective review of cases of interest still often relies on manual analysis of thousands of free-text reports.

Previous studies have leveraged information contained in radiology reports to estimate the frequency of positive findings to assess the appropriate use of imaging for various indications (3–5). Assessing the relevance of imaging is particularly pertinent for advanced and expansive examinations such as MRI and CT in already high-intensity settings, such as on-call shifts. For instance, the use of MRI for emergency department patients with headache is frequent in Europe and increasing in the United States (6). Ongoing debates over the reported overuse of imaging for this indication (7) and the potential severity of subtle cases highlight the need for a large volume of data to address the appropriateness of MRI in this indication. However, the post hoc identification of patient cohorts, as well as the extraction of information from free-text reports, rely on time-consuming human reviews of hundreds of reports.

Readily available methods to automate information extraction from imaging reports to address this question are lacking. Existing solutions rely on either (*a*) rule-based methods requiring language- and institution-specific dictionaries or (*b*) bidirectional encoder representations from transformers (or BERT)–based models dependent on subsequent fine-tuning (8). Although well suited for pretargeted tasks, these methods usually lack the expected flexibility to match the diversity of radiology reports and intended aims; tedious and specific training or adaptation to the institutional data are still necessary (1).

Large language models (LLMs), owing to their training on extensive natural language databases, exhibit unique adaptability to new tasks in different languages (9). Larger models such as OpenAI’s Generative Pre-trained Transformer 4 (GPT-4) and ChatGPT, as well as Google’s Bard (now known as Gemini), have demonstrated excellent performance in various medical tasks without requiring fine-tuning (9). However, their cloud-based nature conflicts with the necessity for data confidentiality, leading to debates regarding their compatibility with real-life medical data (10). A recent study on radiology reports had to create fictitious reports to circumvent this issue (11). Fink and colleagues (12) showed that measurements from previous examinations could be extracted from CT reports with GPT-4, while calling for replication of their findings with privacy-preserving models. In this context, the use of LLMs that are accessible on premises, such as LMSYS Org’s Vicuna (13), could represent a promising alternative. A recent study assessed the ability of Vicuna to extract findings from chest radiograph reports from a publicly available database (14). However, to the best of our knowledge, no study has investigated the use of open-source LLMs for information extraction from real-life radiology reports. The objective of this study was to assess the performance of a task-agnostic, on-premise LLM in information extraction tasks from real-life radiology reports, using human review as the reference standard.

## Materials and Methods

This retrospective study aimed to evaluate the feasibility of automated information extraction from French free-text radiology reports with an open-source LLM. The data warehouse from which the reports were extracted was approved by the French data protection authority (reference no. 2019–103). Use of the data for this specific study was approved by the Lille University Hospital institutional review board in June 2023 (EDS2307251350).

### Data

**Figure 1:**
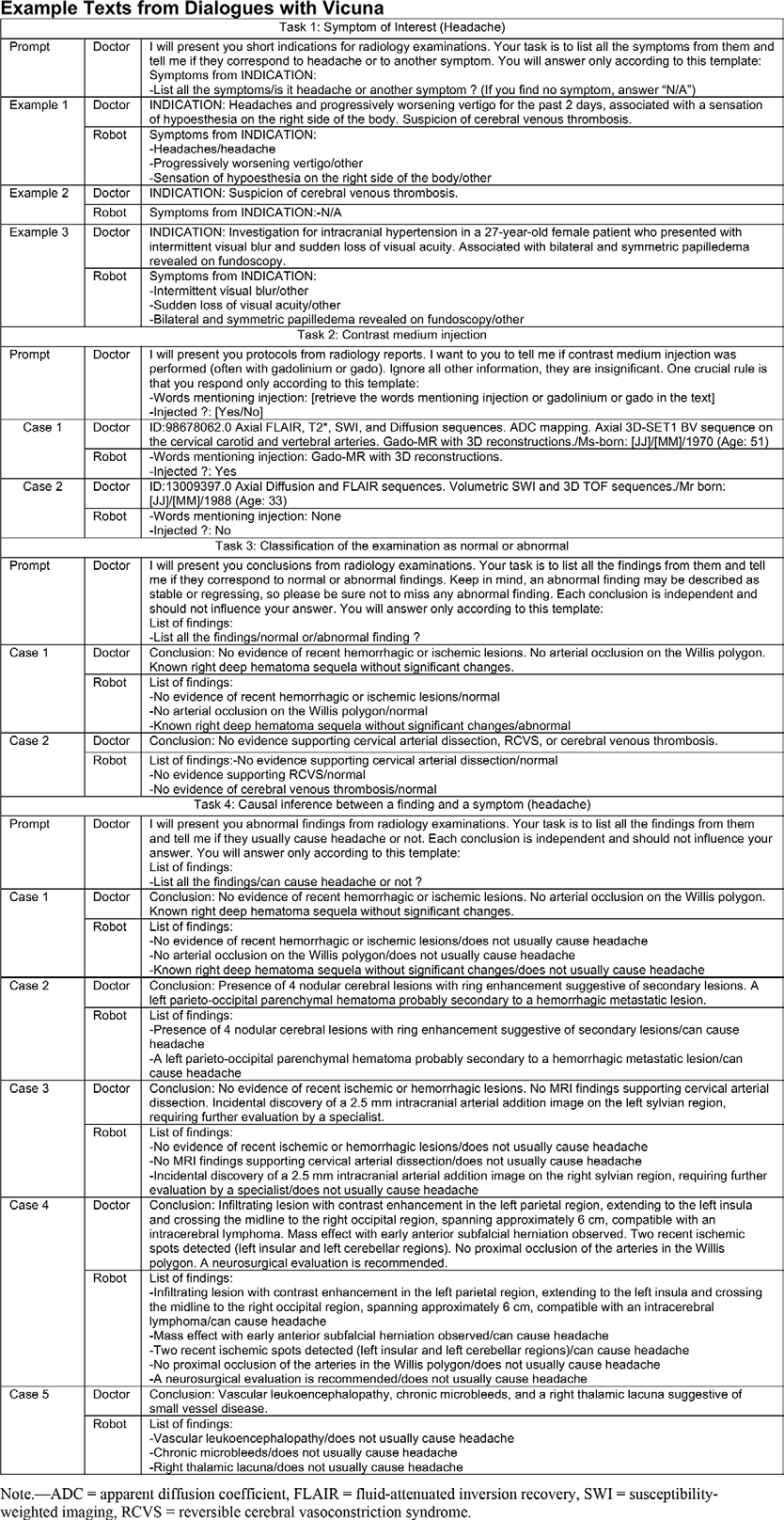
Example texts from dialogues with Vicuna 13-B (LMSYS Org).

Free-text reports were obtained from the health data warehouse of our institution. They were pseudonymized by detecting and removing the place of residence of the patient, their name, and the name of the prescribing physician using eHOP software (Université de Rennes). Eligible reports were brain MRI scans from patients in the emergency department performed from January 2022 to December 2022. Reports were segmented into sections—clinical context, protocol, results, and conclusion—with Python, version 3.10 (Python Software Foundation). The report text itself was left unaltered. Reports were initially written in French and were not translated to English for this study. For reference, texts shown in Figure 1 and Tables 1, S1, and S2 were translated to English by the authors and manually modified for de-identification. Reports were initially written in free text by 22 trainees and 21 board-certified radiologists, who were unaware of this study at the time of reporting.

**Table 1:**
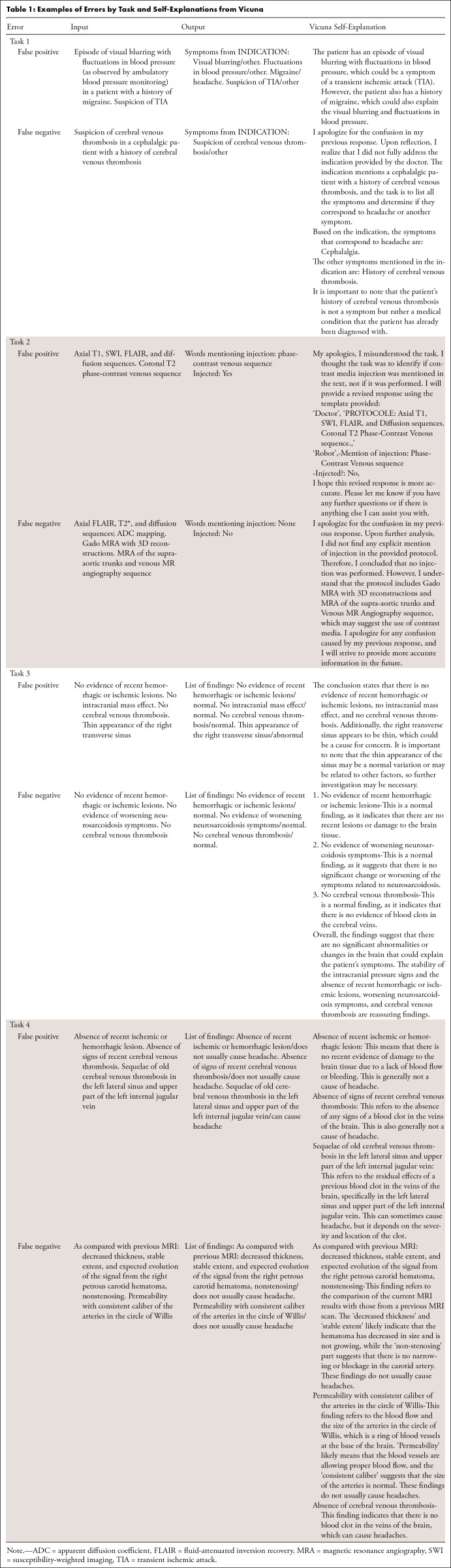
Examples of Errors by Task and Self-Explanations from Vicuna

**Table 2:**
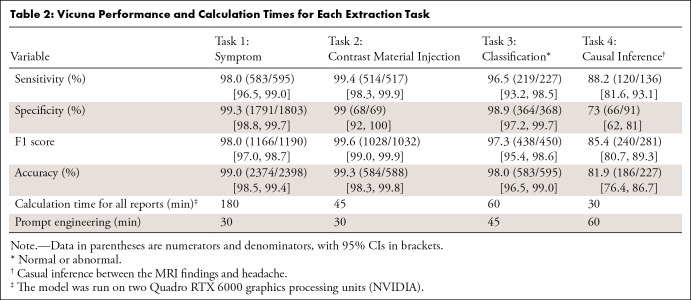
Vicuna Performance and Calculation Times for Each Extraction Task

### Variable Extraction

Four clinically pertinent information extraction tasks were defined as follows: (*a*) presence of headache as a symptom from the clinical context, (*b*) presence of contrast medium injection from the protocol, (*c*) classification of the examination as either normal or abnormal based on its conclusion, and (*d*) inference of causality between the findings from the conclusion and the headache presented by the patient. All information was initially reported by the radiologist as free text. Age and sex of the patients were automatically inserted in the reports by IMPAX Ris Qdoc, version 6.1 (AGFA Healthcare) and were subsequently collected with regular expression matching using Python, version 3.10.

### Ground Truth

Ground truth by task was defined as follows: For tasks 1 and 2, ratings from two in-training radiologists (B.L.G. and A.L., with up to 3 years of experience in neuroradiology) were used. Headache had to be explicitly mentioned in the report to be considered a positive finding. Symptoms had to be acute, recently exacerbated, persistent despite analgesia, or atypical for the patient. For tasks 3 and 4, ratings from two board-certified neuroradiologists (C.B. and G.K., with 7 years and 13 years of experience, respectively) and the two previously involved trainees were used as ground truth. The objective, as initially declared to the raters, was to determine the frequency of findings responsible for headache in patients in the emergency department who underwent a brain MRI examination in our center. Raters had access only to the section of the report related to the task. All ratings were performed independently, with each rater blinded to the ratings of their colleagues and the Vicuna model. Discrepancies were settled by consensus of the two senior radiologists. For task 4, the possible ratings were responsible for headache or not responsible for headache. Then the rater could determine whether they were certain or not certain about their rating. The causal nature of a finding was left to the appreciation and experience of the raters, as done in previous studies (4,5). No predefined list of pathologies was established to account for the specificity of each report, as findings from a single diagnostic group (eg, sinus pathologies) can be considered headache-causing or not depending on the size, shape, signal, enhancement, and adjectives used by the reporting neuroradiologist (as well as potential changes from previous examinations) (4). Consensual examinations were defined as reports for which at least three radiologists rated the association between the main finding and the headache as certain.

### Sample Size Calculation

Sample size was calculated using a previously developed equation (15). To estimate sensitivity and specificity for task 4 of 0.7 (16) with a 95% CI, a precision of 10%, and an expected prevalence of causal findings of 20% (4), the number of reports should be at least 400. With approximately 20% of emergency MRI examinations performed for headache in our institution, at least 2000 reports were screened.

### Model

**Figure 2:**
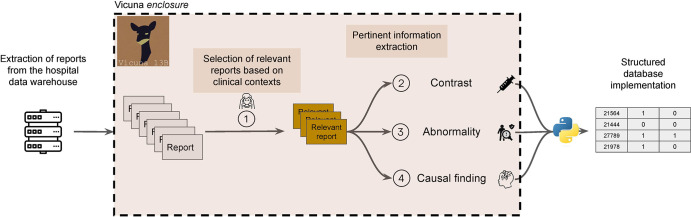
Diagram shows workflow for automated information extraction from pseudonymized radiology reports with Vicuna 13-B (LMSYS Org), an open-source large language model. Four tasks are defined as follows: *1*, reports’ triaging based on the presence of headache; *2*, extraction of contrast medium injection from the protocol; *3*, classification of the examination as either normal or abnormal based on the conclusion; and *4*, causal inference between the main finding and headache.

Version 1.3 of the Vicuna 13-B model was used (*https://huggingface.co/lmsys/vicuna-13b-v1.3*). Vicuna is based on the LLaMA model by Meta and fine-tuned on conversations shared by users on ShareGPT (12). We made no alterations to the model. To mitigate variability, temperature was set to 0. Interaction with the model was made through FastChat (*https://github.com/lm-sys/FastChat*) (17). We developed a Python script to automate interactions with Vicuna and to control the quality of its output (*https://github.com/BastienLeGuellec/RadioVicuna*). The model had access to the same section of the report as the human raters. The final script took free-text reports as input and provided a table as output (Fig 2). The model was run on two Quadro RTX 6000 graphics processing units (NVIDIA).

### Prompting

Prompts were written in English. They were designed to be short and simple to adapt intuitively to new tasks and align with empirical best practices for prompting LLMs (18). Vicuna was specifically prompted to segment its answers to improve the transparency of error analysis (Table 1). We used few-shot in-context learning: fake contextual examples created manually were provided to the model (19). Increasing numbers of examples were used until saturation of diagnostic performance. Examples were engineered to depict the variability of the sample (positive and negative examples, frequent phrasing used by the radiologists). In a sensitivity analysis, we repeated the same tasks with prompts translated from English to French (Tables S3 and S4). For each error displayed in Table 1, we prompted the model to detail its initial rating in a zero-shot approach (no additional example was provided to the model for this prompt): “Reflect on your answer and detail it” (Table 1).

### Performance Evaluation and Statistical Analysis

We estimated sensitivity, specificity, accuracy, and F1 scores, with 95% binomial CIs, against the human-established ground truth. Interrater agreement was measured with Fleiss κ statistics. We tested the robustness of the results with different numbers of contextual examples. For task 4, comparison of the model accuracy between the consensual reports and the rest of the reports was performed with a χ^2^ test. Comparison of performance between prompts in English and French was tested with a McNemar test. *P* < .05 was considered to indicate statistical significance. All statistical analyses were performed by a statistician (A.H.) not involved in the rating of the reports using R software, version 4.3.1 (R Foundation).

## Results

### Radiologist Ratings

**Figure 3:**
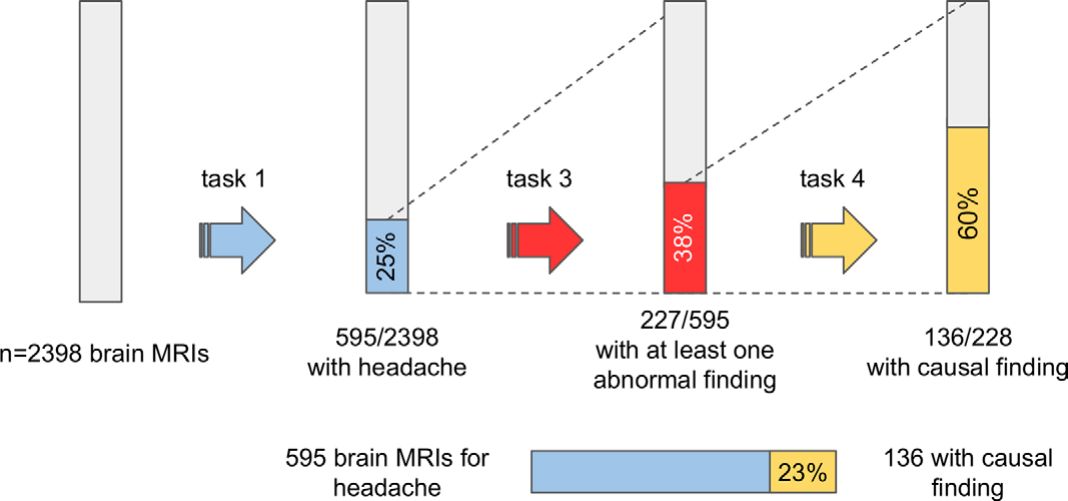
Graph shows diagnostic yield of brain MRI scans in patients in the emergency department with headache.

Among the 2398 brain MRI scans included in the study, radiologists identified 595 scans performed for headache (median age of patients, 35 years [IQR: 26–51 years]; 403 [68%] women and 192 [32%] men). In seven of those reports, contrast medium injection could not be inferred directly from the text, and so those reports were excluded from the task 2 analysis. Of the remaining 588 scans, 517 (88%) were performed with contrast medium injection. Thirty-eight percent (227 of 595) of reports included an abnormal finding in the conclusion (interrater Fleiss κ: 0.96 [95% CI: 0.95, 0.99]). Twenty-three percent (136 of 595) included at least one abnormality that could certainly or probably explain the patients’ headaches (interrater Fleiss κ: 0.77 [95% CI: 0.72, 0.83]) (Figs 3 and S1). Sixteen percent (95 of 595) reported unrelated findings that were not the cause of headache. Diagnoses are available in Table S5. The ranges of the estimated time spent by each radiologist to review and rate the examinations was 180–210 minutes for the 2398 examinations of task 1, 90–105 minutes for the 588 examinations of task 2, and 100–140 minutes for tasks 3 and 4.

### Vicuna Performance

**Figure 4:**
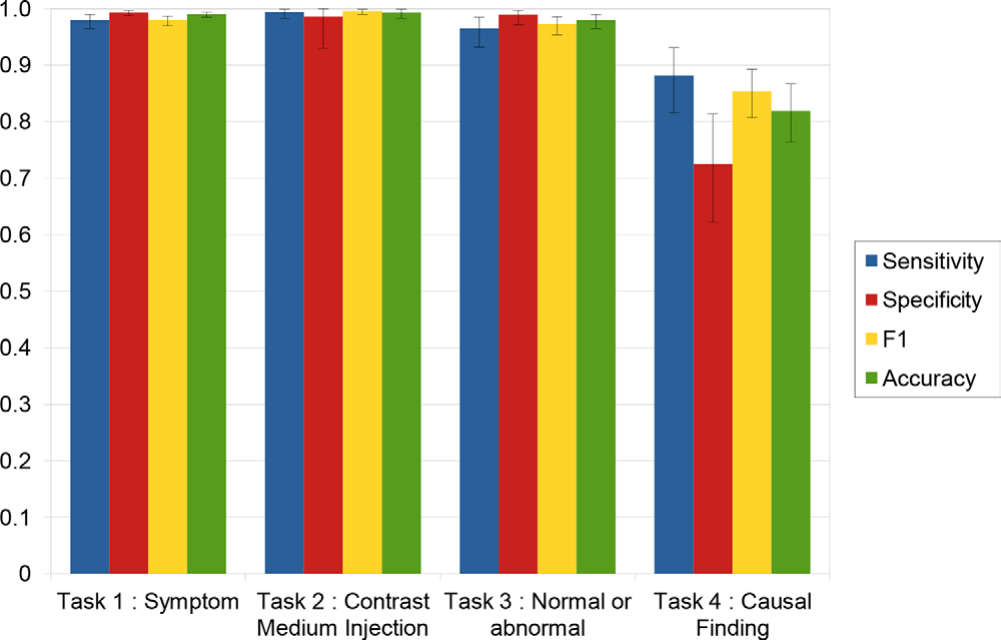
Graph shows performance of Vicuna 13-B (LMSYS Org) for tasks 1, 2, 3, and 4. Error bars are 95% CIs.

All performance metrics were more than 95% for assessment of the presence of headache as a symptom in the indication (sensitivity, 98.0% [95% CI: 96.5, 99.0]; specificity, 99.3% [95% CI: 98.8, 99.7]; accuracy, 99.0% [95% CI: 98.5, 99.4]), contrast medium injection assessment (sensitivity, 99.4% [95% CI: 98.3, 99.9]; specificity, 98.6% [95% CI: 92.2, 100.0]; accuracy, 99.3% [95% CI: 98.3, 99.8]), and classification of the examination as either normal or abnormal based on the conclusion of the report (sensitivity, 96.0% [95% CI: 92.5, 98.2]; specificity, 98.9% [95% CI: 97.2, 99.7]; accuracy, 97.8% [95% CI: 96.3, 98.8]). Causal inference between the main findings of the examination and the patient’s headache as a presenting symptom was accurate in 81.9% (95% CI: 76.4, 86.7) of cases (sensitivity, 88.2% [95% CI: 81.6, 93.1]; specificity, 73.0% [95% CI: 62, 81]) (Table 1 and Fig 4). For this task, Vicuna performed significantly better on consensual examinations (accuracy, 89.7% [95% CI: 82.6, 84.5] on 116 consensual examinations; accuracy, 73.9% [95% CI: 64.7, 81.8] on the remaining 111 reports; *P* = .002) (Table S6). No change in performance metrics was observed when prompting Vicuna in French (Tables S3 and S4).

Prompts were engineered in 30 minutes for tasks 1 and 2 and 1 hour for tasks 3 and 4. Calculation time on our machine ranged from 30 minutes for task 4 (227 reports) to 3 hours for task 1 (2398 reports) (Table 1). Prompts used and examples of answers are provided in Figure 1. Contingency matrices are available in Table S7.

Providing fake contextual examples (few-shot prompting) systematically improved performance over the zero-shot approach. Saturation of accuracy was obtained with four to six examples, depending on the task (Table S8). Contextual examples are provided in Table S1.

### Vicuna Errors Analysis

For all tasks, no systematic error pattern could be identified (Fig 1). False-positive and false-negative errors included various phrasings, contexts, and findings. For task 4, aneurysms were frequently marked as responsible for headache by Vicuna (four false-positive errors), and neither of the two cytotoxic corpus callosum lesions were marked as positive findings (false-negative errors). No other finding was frequently associated with errors. Among the 236 reports on which all four tasks were performed, 49 (22%) had a misclassification for one task, and only one report had a misclassification for two different tasks (Table S9).

When prompted to self-analyze its responses, Vicuna provided the user with additional information or self-corrections, displayed in Figure 1.

## Discussion

An on-premise and open-source LLM extracted information from real-life free-text radiologic reports with excellent accuracy (>95% across three tasks) and performed causal inference between findings and a symptom with 82% accuracy, without requiring specific training.

Human manual review of 2398 brain MRI reports for patients in the emergency department with headache revealed that 25% of emergency MRI examinations were performed for patients with headache, and among those, 23% had significant findings that could explain the headache. A recent meta-analysis (20) reported a rate of causal findings of 9%, significantly lower than ours. That review pooled studies with different imaging modalities (MRI and CT), regions, and center types (secondary, tertiary). Our results are in line with a recent study (4) on brain MRI scans for nontraumatic headache conducted in a Finnish tertiary center, yielding a 20% rate of causal findings.

Performances of commercially available LLMs for feature extraction tasks are still debated (21,22). Initial studies using GPT-3 (OpenAI) on benchmark data reported accuracy below 60% for various name-entity recognition and relation extraction tasks (21). More recent works on GPT-4 reported higher accuracy on simpler tasks, notably more than 90% precision to retrieve medication names from public datasets, improving with the use of self-verification methods (22). Using GPT-4, Adams and colleagues (11) structured fictitious radiology reports with 100% accuracy, and Fink and colleagues (12) successfully extracted measurements from cancer lesions in 96% of reports. Along with these results, we found that a lesser-powered open-source LLM chatbot can extract a symptom from a clinical context and the presence of contrast medium injection from an MRI protocol with near-perfect accuracy. Recent studies emphasize the innate medical knowledge of LLMs and their logical abilities (23,24), which improved with a low number of contextual examples (19). Although the causal inference task described herein relies on the initial inference process of listing findings from images, our results offer insights into a new approach for inferring causality in radiology reports (25).

The ability of open-source models to run on premises has multiple advantages. First, the absence of communication of medical data to private third-party servers ensures critically needed data privacy (10). Second, as opposed to cloud-based methods relying on ever-changing algorithms, reproducibility over time (version control) can be ensured (26). This allows for replicability of results and monitoring model drift during continuous optimization. Third, the training of task-agnostic models on diverse natural texts distinctively separates their intended use as chatbots from their emerging application in medical texts. This separation significantly mitigates the risk of overfitting, as the model’s training data do not overlap with the specific data it encounters in clinical settings. Finally, no additional cost is associated with the use of local models. As recently stated (27,28), the cost per token generated for commercial method scaling with the volume of text to analyze may hamper their widespread use.

The method we propose is based on a publicly available task-agnostic model that was not altered for this study. This approach ensures that the method is easily adaptable for a variety of new tasks across different centers and languages without the need for extensive setup. The model’s comprehensive initial training enables it to effectively manage synonyms, negations, and circumlocutions without the need for further postprocessing, which sets it apart from traditional text-searching techniques. The code for our scripts is made available, and a practical guide is provided (*https://github.com/BastienLeGuellec/RadioVicuna*). Because the model we used is inherently task agnostic, we hypothesize that our method could also be applied to other medical texts beyond radiology reports (29–31).

Explainability of artificial intelligence is a critical aspect of its practical implementation, especially in health care. Our approach allows for direct engagement with the LLM to solicit explanations for each evaluation it provides, ensuring that its answers are based on plausible medical knowledge and correct understanding of the task. Other methods exist to assess the level of certainty for LLM ratings, including examining the probability associated with each output token, a proxy for confidence level (32). Accessing this confidence metric is possible with our proposed method, with detailed instructions available in the practical guide. Future work will need to integrate additional aspects of LLM explainability (33), enhancing transparency and trustworthiness of the proposed workflows.

In the future, LLMs will likely serve as powerful tools for several individuals: (*a*) radiologists, by extracting information from previous examinations to facilitate follow-up (12), drafting reports to improve workflow (34), or checking for readability and completeness of their reports; (*b*) clinicians, by annotating or structuring the report to improve its readability; and (*c*) patients, by providing an assistant able to explain the examination and answer related questions in real time (35). Although recent studies have highlighted the diagnostic abilities of LLMs in the field of radiology (16), LLMs may be integrated sooner into the workflow of radiologists as assistants for low-expertise tasks. This integration is expected to enhance and promote the radiologists’ expertise rather than replace it, relieve them of time-consuming tasks, and facilitate more effective communication of information in their reports. Moreover, from a research perspective, LLMs could substantially contribute to addressing the scarcity of high-quality labeled data for training computer vision models. By leveraging the expert information already present in the report, LLMs could enable a substantial increase in the volume of labeled data, with a limited cost.

This study had limitations. First, because this was a single-center study, we could not test for variability in center writing styles and languages. However, our center is particularly large (165 000 emergency visits annually), and our dataset is diverse with 43 different writers. Because our prompts were in English and reports were in French, we anticipate high robustness to language variations (36), as also evidenced by similar performances after translating the prompts from English to French. Second, the available clinical information was restricted to brief contexts from the report itself. Further studies should extend the scope of this method to data extraction from unsegmented clinical reports. Third, the reference standard for the most complex task of causal inference between a finding and a symptom is in itself subjective, based on the experience of the radiologists. As in previous studies, ground truth was defined by expert consensus (4,5). The difficulty of this task, as shown by the high rate of nonconsensual examinations in our studies (49%), may originate from the frequent lack of explicit comment by the reporting neuroradiologist on the significance of findings. Future works on LLMs, which have shown promise in detailing and simplifying radiology reports (35), may address this issue. Finally, we used a model that will potentially be outperformed by new developments, especially with the recent release of LLaMA 2 (37). However, this workflow is not limited to a specific model or set of models, allowing for flexibility with newer technologies. Further work is needed to study the respective strengths and limits of newer available models.

In conclusion, this proof-of-concept study shows the potential of open-source LLMs to perform information extraction tasks from real-life free-text radiology reports, without the need for additional training. Given the rising social and scientific need for transparency in artificial intelligence, the open-source framework we provide creates a stepping stone for replication studies and may open a new horizon for automated analysis of unstructured medical texts.
